# Bleaching‐Resistant Super‐Resolution Fluorescence Microscopy

**DOI:** 10.1002/advs.202101817

**Published:** 2022-01-27

**Authors:** Jiwoong Kwon, Mohamed Saleh Elgawish, Sang‐Hee Shim

**Affiliations:** ^1^ Department of Biophysics and Biophysical Chemistry Johns Hopkins University Baltimore MD 21205 USA; ^2^ Department of Chemistry Korea University Seoul 02841 Republic of Korea; ^3^ Medicinal Chemistry Department Faculty of Pharmacy Suez Canal University Ismailia 41522 Egypt

**Keywords:** fluorophore, photobleaching, photostability, photoswitching, super‐resolution fluorescence microscopy

## Abstract

Photobleaching is the permanent loss of fluorescence after extended exposure to light and is a major limiting factor in super‐resolution microscopy (SRM) that restricts spatiotemporal resolution and observation time. Strategies for preventing or overcoming photobleaching in SRM are reviewed developing new probes and chemical environments. Photostabilization strategies are introduced first, which are borrowed from conventional fluorescence microscopy, that are employed in SRM. SRM‐specific strategies are then highlighted that exploit the on–off transitions of fluorescence, which is the key mechanism for achieving super‐resolution, which are becoming new routes to address photobleaching in SRM. Off states can serve as a shelter from excitation by light or an exit to release a damaged probe and replace it with a fresh one. Such efforts in overcoming the photobleaching limits are anticipated to enhance resolution to molecular scales and to extend the observation time to physiological lifespans.

## Graphical abstract

In super‐resolution fluorescence microscopy, photobleaching not only leads into gradual loss of fluorophores but also greatly influences on spatiotemporal resolution. Both conventional photostabilization strategies applied in super‐resolution imaging and specific approaches repurposing fluorescence‐off states as shelters, are reviewed. Off states, originally devised for overcoming the diffraction limit, can shelter fluorophores from excitation or for replacing to a new one.

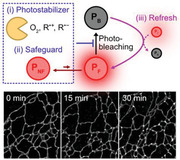

## 1 Introduction

The recent technical maturation of super‐resolution microscopy (SRM) has revealed ultrastructures inside living cells with dynamics that are far beyond the diffraction limit.^[^ ^1^, ^2^, ^3^, ^4^, ^5^, ^6^, ^7^, ^8^, ^9^ ^]^ The performance of SRM is often influenced by the photophysical properties of the fluorophores used, including switching characteristics and photobleaching resistance. Bleaching‐resistant fluorescent probes are especially important to ensure long‐term imaging capabilities as well as higher spatiotemporal resolution. Numerous efforts on bleaching‐resistant probes and their applications for SRM have been made to further improve the performance of SRM.

### 1.1 The Evolution of Super‐Resolution Microscopy

Fluorescence microscopy has been an important and powerful tool for biological research because it is noninvasive for living cells and has excellent molecular specificity.^[^ ^10^ ^]^ The unique benefits of fluorescence microscopy enable the acquisition of the three‐dimensional structures, locations, and distributions of specific molecules of interest in fixed and living specimens. Yet the spatial resolution of fluorescence microscopy is restricted by the limit of diffraction (*s* ≈ *λ*⁄2*NA*, where *s* is the spatial resolution, where *λ* is the wavelength of light, and *NA* is the numerical aperture of the microscope objective), which hinders accurate measurements on biomolecules with average sizes on the nanometer scale.

In the 1990s, developments in optics and electronic devices greatly enhanced the sensitivity of fluorescence detection, permitting the detection of single molecules even at room temperature.^[^ ^11^, ^12^ ^]^ Meanwhile, strategies to break the diffraction limit were suggested by Stefan W. Hell and coworkers,^[^ ^13^, ^14^, ^15^ ^]^ and subsequent experiments demonstrated sub‐diffraction limit resolution in the lateral and axial directions.^[^ ^16^, ^17^ ^]^ In the 2000s, the development of photoactivatable and photoswitchable fluorescent probes provided additional means to overcome the diffraction limit via single‐molecule localization.^[^ ^18^, ^19^, ^20^, ^21^, ^22^, ^23^, ^24^, ^25^, ^26^ ^]^ Moreover, patterned illumination was used to retrieve high spatial frequency information from moiré fringes that led to two‐fold enhancements in spatial resolution.^[^ ^27^, ^28^ ^]^ These pioneering attempts over the past two decades enabled the advent of diverse super‐resolution techniques that are now commonly employed to probe biological phenomena at the molecular level.^[^ ^3^, ^4^ ^]^

### 1.2 Fluorescence Transitions As a Core Mechanism of Super‐Resolution Microscopy

**Figure 1:**
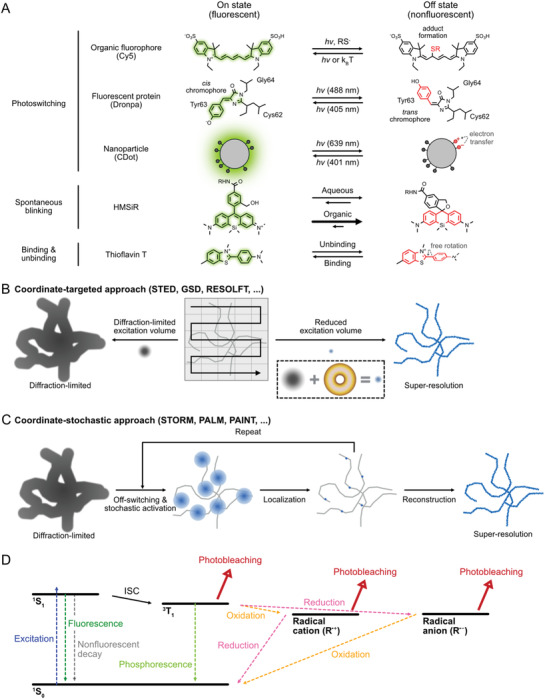
Fluorophores, principles and photobleaching pathways in super‐resolution microscopy (SRM). A) Schematic illustration of fluorescence on–off transitions utilized in SRM. Representative examples are selected for the light‐induced transitions (first three rows), a spontaneous transition (fourth row) and a binding‐mediated transition (fifth row). B) Coordinate‐targeted approaches for SRM. On‐ and off‐transitions occur only on designed locations to reduce the effective emission volume. C) Coordinate‐stochastic approaches for SRM. A small, random fraction of molecules turn on at a given camera frame, allowing precise single‐molecule localization. D) Energy diagram of photophysics including possible photobleaching pathways for organic fluorophores.

The majority of super‐resolution techniques, specifically those with theoretically unlimited resolution, utilize the nonlinear fluorescence responses with respect to the intensity of excitation light.^[^ ^29^ ^]^ The nonlinearity often originates from the on‐ and off‐transitions of fluorescence (**Figure** **1**A). Since the population in off states does not emit fluorescence, the bulk fluorescence intensity is not linear to the excitation intensity. The off state can be a nonfluorescent state or a spectrally different state. Saturation of fluorescence also contribute to nonlinearity and further enhances spatial resolution.

Super‐resolution techniques are categorized into two different groups depending on how the nonlinear response is exploited. The first group induces fluorescence transitions only in the targeted region and is frequently referred to as “coordinate‐targeted” approaches (Figure 1B).^[^ ^30^ ^]^ Stimulated emission depletion (STED),^[^ ^14^ ^]^ ground state depletion (GSD),^[^ ^15^ ^]^ reversible saturable optical transition (RESOLFT),^[^ ^31^ ^]^ and (saturated/nonlinear) structured illumination microscopy (SIM)^[^ ^27^, ^32^ ^]^ are representative techniques in this first “coordinate‐targeted” category. These approaches reveal the high‐frequency information from the spatially and temporally manipulated fluorescence patterns. Saturation of the fluorescence transition plays a key role in resolution improvement because highly saturated transitions provide higher spatial frequency information and thus better spatial resolution. By comparison, the second group is termed as “coordinate‐stochastic” approaches, which operate from fluorescence transitions that occur randomly over a wide observation area (Figure 1C).^[^ ^30^ ^]^ (Direct) stochastic reconstruction microscopy ((d)STORM),^[^ ^23^, ^33^ ^]^ (fluorescence) photoactivatable localization microscopy ((F)PALM),^[^ ^24^, ^25^ ^]^ ground state depletion microscopy followed by individual molecule return (GSDIM),^[^ ^34^ ^]^ and point accumulation for imaging in nanoscale topography (PAINT)^[^ ^26^ ^]^ are involved in this second category. Coordinate‐stochastic approaches exploit the precise localization of stochastically observable single fluorophores to reconstruct a high‐resolution image.^[^ ^19^ ^]^ Thus, such approaches are more generally called “single‐molecule localization microscopy (SMLM)” methods. The population equilibrium between on‐ and off‐states and detectable photon numbers from single molecules in each frame are the predominant determinants of the spatial resolution in SMLM. Shortly after the initial demonstration of the super‐resolution concepts, super‐resolution techniques rapidly advanced to provide multicolor, multidimensional, and dynamic information of biomolecules from living specimens at up to single‐digit (<10 nm) spatial resolutions.

Fluorescence transitions between on‐ and off‐states are core frameworks of SRM, and different switching mechanisms are exploited by different techniques. Many of the first super‐resolution techniques used light‐mediated fluorescence transitions (i.e., photoswitching) due to the convenient control on switching rates. For example, STED and GSD/GSDIM microscopies employ singlet ground state (^1^S_0_) and first excited triplet state (^3^T_1_) as light‐inducible nonfluorescent states, respectively. RESOLFT/(d)STORM/(F)PALM used photoreactions to switch on and off fluorophores.^[^ ^17^, ^23^, ^24^, ^25^, ^34^, ^35^, ^36^ ^]^ Subsequently, binding‐mediated and spontaneous transitions are used in SRM to minimize unwanted photodamage to the fluorophores, as well as biological samples.^[^ ^26^, ^37^, ^38^ ^]^ Currently, the rational design of fluorophores with switchable activities has emerged as an important field for enhancing performance in SRM.^[^ ^39^, ^40^, ^41^ ^]^

Recent advances in SRM developed many different strategies to break the diffraction limit, and a significant fraction of them is not well categorized into above two groups. For example, fluctuation‐based approaches such as super‐resolution optical fluctuation imaging (SOFI) and super‐resolution radial fluctuation (SRRF) are close to “coordinate‐stochastic” approach, but they achieve sub‐diffraction‐limit resolution by totally different mechanisms to the single‐molecule localization.^[^ ^42^, ^43^ ^]^ Expansion microscopy physically expands the sample by up to 10 times and allows super‐resolution image with conventional microscopes, so it is distinct from the two categories.^[^ ^44^ ^]^ These strategies provide alternative options that can be chosen depending on the samples and imaging conditions to investigate nanostructures.

### 1.3 Photophysics and Image Quality in Various Super‐Resolution Techniques

Each super‐resolution approach requires fluorophores with different properties, specifically the photophysical characteristics, which have a strong bearing over the resulting quality of the imaging. For example, fluorophores with large Stokes shifts are commonly used in STED approaches, especially in multicolor applications, to maximize the cross‐section of the stimulated emission.^[^ ^45^ ^]^ Fluorophores with high resistances to photobleaching permit higher depletion intensities and greater numbers of fluorescence transition cycles, which ultimately promote higher spatial resolution.^[^ ^46^ ^]^ In SMLM, fluorophores with high photon numbers and low duty cycles are required to obtain the best image quality.^[^ ^47^ ^]^ Generally, super‐resolution techniques require fluorophores that support fast transition rates to nonfluorescent states and high resistance to photobleaching. Photobleaching is a complex phenomenon that involves multiple pathways and is still not fully understood (Chapter 1.4). As different SRM techniques use intense light with different wavelengths (i.e., SMLM employs wavelengths near the maxima of the absorption spectra, whereas STED operates the depletion laser at the tail of the emission spectra), their detailed photobleaching pathways may be different. Modality‐specific photostabilizing strategies might be needed to achieve the best anti‐bleaching performance in SRM techniques.

The rationale for the requirements of fluorophores for each super‐resolution technique are summarized below.^[^ ^39^ ^]^ These discussions for relating photophysics and image quality is to give rationale on how photobleaching‐resistance affects the image quality of each technique.

1) STED, GSD, and RESOLFT
  – Operating principle: STED and related methods achieve sub‐diffraction limit resolution by depleting peripheral fluorescence around the focal spot (Figure 1B). This is achieved by using a doughnut‐shaped depletion laser for the off‐switching transition that effectively reduces the point spread function (PSF). The spatial resolution of these approaches depends on the size of the effective PSF (*x*) and the Nyquist resolution (*d*) from the scanning step size, by following relationships
(1) $$x\approx \frac{s}{\sqrt{1+I/I_{s}}}$$
    where *s* is the size of the diffraction‐limited PSF, *I* is the applied depletion intensity, and *I_s_* is the characteristic saturation intensity of the fluorophore in use
(2) $$d\approx \frac{\lambda }{NA\sqrt{N_{\mathrm{cycles}}}}$$
    where *λ* is the wavelength of light, *NA* is the numerical aperture, and *N_cycles_* is the number of switching cycles. Based on Equations (1) and (2), for example, a 5‐fold higher spatial resolution can be achieved with a STED microscope only when the fluorophore can survive at least 25 on–off switching cycles under irradiation with a depletion intensity that is ≈25 times higher than *I_s_*.
  – High off‐switching rate: The saturation intensity (*I_s_*) is a characteristic value for these approaches that refers to the point at which the fluorescence intensity is depleted to 50% from an initial level. High off‐switching rates correspond to lower *I_s_* values, and thus better spatial resolution (*x*) at a specified depletion intensity (*I*). The stimulated emission cross‐section, intersystem crossing rate, and cross‐section of the light‐induced transition to a nonfluorescent state are key determinants of the off‐switching rate in STED, GSD, and RESOLFT approaches, respectively.
  – High resistance to photobleaching: Photobleaching is the permanent loss of fluorescence of a fluorophore, which typically arises from photoreactions that are mediated by the excited state. Photobleaching‐resistant fluorophores permit higher depletion intensities that promote better spatial resolution (*x*), allow the use of increased numbers of switching cycles (*N_cycles_*), and extended observation times. Additional switching cycles reduce the scanning step size (i.e., pixel size) and enhance Nyquist resolution (*d*).
2) SMLM
  – Operating principle: In SMLM, most fluorophores remain in a nonfluorescent state, while a very small fraction (≪1%) are stochastically switched on to a fluorescent state such that diffraction‐limited spots are resolvable from one another in each camera frame. Each spatially separated molecule has a Gaussian‐like PSF with a center position that is accurately estimated by using a 2D Gaussian fit on the signal from a single molecule. The precision of the single‐molecule localization process (*σ*) is affected by several factors including the PSF width (*s*), pixel size (*a*), background noise (*b*), and number of collected photon per frame (*N*) as follows
(3) $$\sigma =\sqrt{\frac{s^{2}}{N}+\frac{a^{2}}{12N}+\frac{8\pi s^{4}b^{2}}{a^{2}N^{2}}}$$
    where *N* is a characteristic photon number of a fluorophore.^[^ ^19^, ^48^ ^]^ The *σ* is also influenced by the optical system and imaging buffer conditions. Fluorophores with high *N* values are commonly used to provide greater spatial resolution for SMLM. Alexa Fluor 647, the most common fluorophore for SMLM, provides ≈5000 photons per switching cycle that corresponds to 10–20 nm of spatial resolution depending on experimental setup.
  – Low on–off duty cycle: The duty cycle (DC) is the fraction of molecules in fluorescent state at equilibrium, which depends on the on‐ and off‐switching rates (*k_on_* and *k_off_*, respectively) as follows
(4) $$\mathrm{DC}=\frac{P_{F}}{P_{F}+P_{NF}}=\frac{1}{1+k_{\mathrm{off}}/k_{\mathrm{on}}}$$
    where *P_F_* and *P_NF_* are the populations of the fluorescent and nonfluorescent states, respectively. Most fluorophores used for SMLM have low DC less than 0.001.^[^ ^47^ ^]^ Lower DC values minimize the overlap between stochastically activated fluorophores and promote high localization densities (LD) that provide better Nyquist resolution (*d*)
(5) $$d\approx 2/LD^{1/C}$$
    where *C* is two for 2D images and three for 3D images.
  – High resistance to photobleaching: SMLM also employs intense laser light that facilitates photobleaching, to collect more photons with a higher frame rate and to reduce the DC. Thus, fluorophores with high resistance to photobleaching offer both higher spatiotemporal resolution and extended observation times for SMLM.

In summary, off‐switching transition rates and photobleaching resistance are important photophysical parameters that influence performance in super‐resolution techniques. In addition, a strong photobleaching resistance often ensures a higher off‐switching rate, owing to its dependence on the light intensity. For live cell applications, these light dose‐dependent photophysical parameters should be carefully tuned to minimize the phototoxicity. Different techniques exploit distinct nonfluorescent states and therefore have distinct requirements for the off‐switching transition properties of fluorophores. For example, spontaneous blinking dyes such as HMSiR are suitable for targets in lipid environments, and can provide low duty cycle without intense light illumination. For nearly all approaches, robust resistance to photobleaching provides greater super‐resolution image quality and enables extended observation times; exceptions are bleaching‐mediated super‐resolution approaches such as single‐molecule high‐resolution imaging with photobleaching (SHRImP).^[^ ^49^ ^]^

### 1.4 Photobleaching Mechanisms and Preventing Strategies

The most common fluorophore family for SRM are synthetic organic dyes, which are small chemicals with highly conjugated structures that promote photo‐absorption to result in the spontaneous emission of fluorescence at high quantum yields. Photobleaching of organic fluorophores is the irreversible transition of the molecule to a non‐emissive state and is considered to occur via a multi‐step process,^[^ ^50^ ^]^ which involves multiple pathways and begins from the excited states (Figure 1D).^[^ ^51^ ^]^ Photobleaching reactions of organic fluorophores are described by two major pathways.^[^ ^52^ ^]^ The first pathway occurs in the presence of dissolved oxygen nearby the organic fluorophore that converts to a reactive singlet oxygen (^1^O_2_) via triplet‐triplet annihilation. Singlet oxygen can either directly oxidize the fluorophore, resulting in photobleaching, or further generate reactive oxygen species (ROSs) such as hydroxyl radicals (OH˙), peroxyl radicals (RO_2_˙), and superoxide anions (O_2_˙^−^).^[^ ^53^ ^]^ ROSs also contribute to photobleaching by reactions involving free radicals. Thus, the removal of dissolved oxygen from the local environments of organic fluorophores significantly reduces the rate of photobleaching.^[^ ^54^ ^]^ Singlet oxygen is considered the major species that induces photobleaching, and the triplet state of the fluorophores is regarded as a precursor to photobleaching. Yet scavenging dissolved oxygen or inhibiting the transition to the triplet state does not completely prevent fluorophores from photobleaching, implying the presence of another photodegradation pathway.^[^ ^55^ ^]^ This second photobleaching pathway occurs via reactive, short‐lived radical intermediates that are produced by the excited‐state‐mediated photoionization (Figure 1D).^[^ ^52^ ^]^ Higher excited singlet (^1^S_n_) and triplet (^3^T_n_) states are closely related to the rate of photoionization. Transitions to triplet states (^3^T_1_ and ^3^T_n_) facilitate electron transfer reactions and induce the irreversible degradation of fluorophores via radical formation. As a result, various combinations of reducing agents, antioxidants, and triplet state quenchers have been proposed to suppress the generation of free radicals and prevent oxygen‐independent photobleaching.^[^ ^56^ ^]^

The discovery of fluorescent proteins (FPs) permitted fluorescent measurements in live cells, largely arising from the capability to easily tag FPs to intracellular protein targets.^[^ ^57^ ^]^ Several amino acids in FPs form a fluorescent chromophore in a stable and structured protein scaffold.^[^ ^58^ ^]^ The FP chromophore is tightly enclosed in the protein, which prevents physical interactions among the chromophore and surrounding solute molecules and therefore makes the photobleaching mechanism distinct from organic dyes.^[^ ^59^ ^]^ The protein scaffold also interacts strongly with the chromophore, typically by direct covalent bonds or multiple hydrogen bonds, to provide conformational stability to the chromophore.^[^ ^60^ ^]^ Supplementary photostabilizing chemicals usually do not affect the photobleaching characteristics of the FP. Photobleaching in FPs is a complicated process that is sensitive to a variety of conditions, including incubation medium and cellular compartment.^[^ ^61^ ^]^ Although many FPs are photostable enough for long‐term live‐cell imaging, some advancements in photostability are still imminent.^[^ ^61^ ^]^ Mutagenesis approaches that introduce point mutations on residues at and/or nearby the chromophore alter photophysical characteristics of FPs, including photobleaching properties.^[^ ^62^, ^63^ ^]^

Developments in nanomaterial technology provided a new class of nanometer‐sized inorganic fluorophores called fluorescent nanoparticles. Fluorescent nanoparticles can have distinct structural and electric configurations and support various emission mechanisms than organic dyes. For example, semiconductor quantum dots (QDots) support quantum confinement effects that allow the emissive relaxation of the excited electrons that promote broad absorption range with large cross‐sections, narrow emission band, and high photostability.^[^ ^64^ ^]^ The photophysical properties of QDots are highly dependent on the QDot structure (i.e., core‐shell) and size. Negatively charged nitrogen‐vacancy (NV^−^) centers in diamonds have a high resistance to photobleaching.^[^ ^65^ ^]^ NV^−^ centers are perfectly encapsulated in the well‐ordered tetrahedral arrays of carbon, which do not allow excited state reactions during fluorescence cycles. As a result, environmental conditions normally do not affect the fluorescence properties of NV^−^ centers. Similarly, the photophysical properties of nanoparticle‐based fluorescent probes are generally controlled by the composition of the particles rather than the environmental conditions.

In this progress report, we summarize the strategies to avoid or overcome photobleaching for various fluorescent probes during super‐resolution imaging. These strategies aim both to extend the observation time and increase the photon budget before photobleaching, in order to improve the performances of SRM techniques. We categorize the bleaching‐resistant strategies into three groups: 1) photostabilization as in conventional fluorescence microscopy; 2) sheltering from excitation to off states; 3) replacement of damaged fluorophores with fresh, new ones (**Table** **1**). Along the line, we introduce recent applications of super‐resolution techniques under bleaching‐resistant conditions to illustrate the benefits of bleaching‐resistance of fluorescent probes to the resultant super‐resolved images. As a guideline, we summarized the photobleaching preventing strategies that will be discussed in this review, by categorizing them for different imaging modalities and fluorescent probes in **Table** **2**.

**Table 1:** Summary on the mechanisms, advantages, disadvantages, and the live‐cell compatibility of the photobleaching preventing strategies

Photobleaching preventing strategies		Preventing mechanism	Advantages	Disadvantages	Live‐cell compati‐bility	References for SRM applications^b)^
Photostabilizing buffers (Chapter 2.1)	GLOX	Scavenging dissolved oxygen	Standard buffer Aqueous environment without ROSs	Acidification	Compatible under limited conditions^c)^	^[^ ^23^, ^33^, ^47^, ^66^, ^67^ ^]^
	PCA/PCD		Higher stabilizing efficiency	Acidification	Compatible under limited conditions^c)^	^[^ ^68^ ^]^
	PYOX		No acidification	Cost‐inefficient	Incompatible	N.A.
	MB‐thiol		No acidification	MB aggregates	Incompatible	^[^ ^69^ ^]^
	OxEA		No acidification Live‐cell and multicolor applications	Less stabilizing efficiency	Compatible	^[^ ^70^, ^71^ ^]^
	Sulfite buffer		No acidification Available for high NA medium Long‐term storage at RT	No peer‐reviewed references yet for SRM applications	Incompatible	^[^ ^72^ ^]^
	D_2_O	Perturbing excited‐state reactions and hydrogen bonds	Stabilizing both organic fluorophores and FPs	Affecting other chemical reactions	Compatible under limited conditions^c)^	^[^ ^73^, ^74^, ^75^ ^]^
Self‐healing dyes (Chapter 2.2)		Intramolecular stabilization by PSG	Efficient stabilizing by intramolecular redox reactions	Internal PSG affects the photophysics	Compatible with additional engineering^d)^	^[^ ^76^ ^]^
Encapsulation (Chapter 2.3)		Physical blocking of dissolved oxygen by forming host‐guest complex	Simple modification	Increase in effective size of dyes Hinder approach of switching reagents	Compatible with additional engineering^d)^	^[^ ^34^, ^77^ ^]^
Structural modifications (Chapter 2.4,5)	Organic fluorophores	Tuning LUMO level by introducing EWG	Small structural change for much higher photostability	Require an expertise on organic synthesis	Compatible with additional engineering^d)^	^[^ ^78^, ^79^, ^80^, ^81^ ^]^
	FPs	Mutagenesis	Most efficient way for more photostable FPs	Require an expertise on protein mutation	Compatible	^[^ ^82^ ^]^
Cryogenic SRM (Chapter 2.6)		Inefficient photobleaching pathways under cryogenic condition	Near‐native biological structures Available for CLEM	Low NA Restricted light intensity Perturbation on the switching kinetics	Incompatible	^[^ ^83^, ^84^, ^85^, ^86^, ^87^, ^88^, ^89^, ^90^ ^]^
Nanoparticles (Chapter 2.7)	QDot	Intrinsic high photostability	Versatility Well‐known chemistry for bioconjugation Commercially available	Relatively weaker photostability than other nanoparticles	Compatible^e)^	^[^ ^42^, ^91^, ^92^, ^93^, ^94^, ^95^ ^]^
	FND (NV^−^ center)		Extreme photostability Commercially available	Large size Limited in multiplexing Difficulties in bioconjugation	Compatible^e)^	^[^ ^96^, ^97^, ^98^, ^99^ ^]^
	CDot		Small size Cost‐efficient Versatility Easy conjugation on biomolecules	Limited in multiplexing	Compatible	^[^ ^100^, ^101^, ^102^, ^103^ ^]^
	UCNP		High STED efficiency	Less stability due to high surface charge	Compatible^e)^	^[^ ^104^, ^105^, ^106^ ^]^
	PDot		Superior brightness Easy conjugation on biomolecules	Restricted in live‐cell applications	Incompatible	^[^ ^107^, ^108^ ^]^
	AIE		High STED efficiency Extreme photostability	Large size	Compatible	^[^ ^109^, ^110^, ^111^ ^]^
Utilizing nonfluorescent state (Chapter 3)	Protective STED^a)^	Multiple off‐switching transitions	Extended observation time by purely optical manner	Technical complexity Require photoswitchable probes for STED imaging	Compatible	^[^ ^112^ ^]^
	Utilizing FRET	Competition between FRET and photobleaching pathways	One of the simplest methods to increase photostability	Decreased brightness due to FRET Less compatible for multiplexing	Compatible	^[^ ^113^ ^]^
	Spontaneously blinking fluorophores^a)^	Spontaneous intramolecular cyclization to form a dark state	Ready‐to‐use probes for SMLM with high photostability	Require chemical engineering Environment‐dependent equilibrium	Compatible	^[^ ^38^, ^114^, ^115^, ^116^, ^117^, ^118^ ^]^
	Utilizing dimer formation^a)^	Distinct spectral property of dimer	Ready‐to‐use probes for SMLM with high photostability	Restricted to BODIPY dye Less compatible for multiplexing	Compatible	^[^ ^119^ ^]^
	Chemical caging of FPs^a)^	Chemical‐induced dark state formation of FPs	Simple way to increase photostability of FP	Low photon numbers for SMLM	Compatible	^[^ ^120^, ^121^ ^]^
Replacing bleached probes (Chapter 4)	Exchangeable organic fluorophores^a)^	Binding‐unbinding equilibrium with pseudo‐infinite pool of probes	Direct usage of probes without additional modifications	Probe‐dependent target restriction High background from unbound probes	Compatible	^[^ ^26^, ^122^, ^123^, ^124^, ^125^, ^126^ ^]^
	DNA‐PAINT^a)^		Controllable binding kinetics Highly multiplexed imaging Accurate quantitative imaging High photon number per switching cycle	High background from unbound probes Slow imaging speed Restriction to live‐cell imaging	Incompatible	^[^ ^127^, ^128^, ^129^, ^130^, ^131^, ^132^, ^133^, ^134^, ^135^, ^136^, ^137^, ^138^ ^]^
	RNA PAINT^a)^		Visualizing specific RNA in live cells	Restricted to RNA	Compatible	^[^ ^139^ ^]^
	Peptide PAINT^a)^		Visualizing specific protein in live cells	Constrained by the number of pairs of peptide‐protein interactions	Compatible	^[^ ^140^, ^141^, ^142^ ^]^
	Protein PAINT^a)^		Visualizing specific protein in live cells	Chemical additives	Compatible	^[^ ^143^, ^144^, ^145^, ^146^ ^]^

^a)^ These photobleaching‐preventing strategies are specifically applicable in SRM, whereas the strategies not marked with a superscript letter can also be employed in common fluorescence imaging applications

^b)^ We only summarized the previous works that are already included in our review. Since there are many studies using SRM, one may be able to find some other examples for even wider applications

^c)^ These are sometimes used for the live‐cell SRM imaging under limited conditions (i.e., short enough observation time around 1 h)

^d)^ Chemically‐modified organic fluorophores can be used for the live‐cell imaging with an additional engineering for the membrane permeability and for the site‐specificity

^e)^ Live‐cell capability of these nanoparticles has been demonstrated mostly for the single‐particle tracking studies that are not discussed in this review.

**Table 2:** Summary of strategies for preventing photobleaching discussed in this review in terms of different imaging modalities and fluorescent probes

Imaging modality	STED/ RESOLFT/ GSD	2.1. Photostabilizing buffers 2.2. Self‐healing dyes 2.4. Structural modifications of organic dyes 2.6. Cryogenic super‐resolution fluorescence microscopy 2.7. Nanoparticles in super‐resolution microscopy 3.1. Protected STED nanoscopy by the use of photoswitchable probes 3.3. Spontaneously blinking fluorophores 4.6. Exchangeable probes for coordinate‐targeted microscopy 4.7. Exchangeable probes for DNA imaging
	SIM	2.5. Structural modification of fluorescent proteins 2.6. Cryogenic super‐resolution fluorescence microscopy
	SOFI	2.6. Cryogenic super‐resolution fluorescence microscopy 2.7. Nanoparticles in super‐resolution microscopy
	SMLM	2.1. Photostabilizing buffers 2.2. Self‐healing dyes 2.3. Encapsulation of fluorophores 2.6. Cryogenic super‐resolution fluorescence microscopy 2.7. Nanoparticles in super‐resolution microscopy 3.2. FRET enhanced photostability 3.3. Spontaneously blinking fluorophores 3.4. Dimerized dyes from a lasting source of monomer 3.5. Chemical caging of fluorescence protein 4.1. Surface PAINT 4.2. DNA‐PAINT 4.3. RNA PAINT 4.4. Peptide PAINT and LIVE‐PAINT 4.5. Protein PAINT 4.7. Exchangeable probes for DNA imaging
Fluorescent probes	Organic dye	2.1. Photostabilizing buffers 2.2. Self‐healing dyes 2.3. Encapsulation of fluorophores 2.4. Structural modifications of organic dyes 2.6. Cryogenic super‐resolution fluorescence microscopy 3.2. FRET enhanced photostability 3.3. Spontaneously blinking fluorophores 3.4. Dimerized dyes from a lasting source of monomer 4.1. Surface PAINT 4.2. DNA‐PAINT 4.3. RNA PAINT 4.4. Peptide PAINT and LIVE‐PAINT 4.6. Exchangeable probes for coordinate‐targeted microscopy 4.7. Exchangeable probes for DNA imaging
	Fluorescent protein	2.5. Structural modification of fluorescent proteins 3.1. Protected STED nanoscopy by the use of photoswitchable probes 3.2. FRET enhanced photostability 4.4. Peptide PAINT and LIVE‐PAINT 4.5. Protein PAINT
	Nanoparticle	2.7. Nanoparticles in super‐resolution microscopy

## 2 Conventional Photostabilizing Strategies Applied in Super‐Resolution Microscopy

As discussed in Chapter 1, different types of fluorescent probes require distinct strategies to improve their resistance to photobleaching. Organic fluorophores are the most common probe molecules that are used in SRM, and photobleaching of these species occurs via reactive species in the environment. The photophysical properties of FPs and nanoparticles are less dependent on surrounding conditions than organic dyes, and the photostabilities of these entities are instead controlled by the internal modifications. In this chapter, we will describe conventional strategies to prevent photobleaching of fluorescent probes and their merits in super‐resolution imaging.

### 2.1 Photostabilizing Buffers

Organic dyes are widely employed as fluorescent tags because they offer several intrinsic advantages, namely small size, brightness, and facile chemical modification. Unfortunately, organic dyes are relatively vulnerable to photobleaching because they are exposed to reactive chemicals in the environment (Figure 1D). The most common approach to prevent photobleaching of organic fluorophores is to remove dissolved oxygen from the imaging environment. Lower amounts of dissolved oxygen induce undesired photoblinking of fluorophores as the triplet oxygens act as an efficient triplet quencher.^[^ ^147^ ^]^ Supplementary small thiol molecules such as *β*‐mercaptoethanol (*β*ME) can restore the fluorophores from severe photoblinking.^[^ ^148^ ^]^ Enzyme‐based oxygen scavenging systems provide an aqueous environment without ROSs and thus have become widely used for a variety of fluorescence measurements, such as fluorescence imaging and single‐molecule spectroscopy, under physiological conditions.^[^ ^149^, ^150^ ^]^ Similarly, SRM techniques under aqueous conditions also benefit from enzymatic oxygen scavenging systems for enhancing spatial resolution.^[^ ^23^, ^33^ ^]^

**Figure 2:**
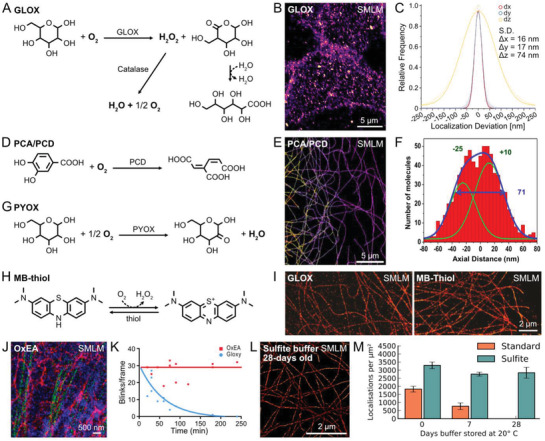
Photostabilizing buffers in super‐resolution microscopy. A) Molecular mechanism of oxygen scavenging in photostabilizing buffers based on glucose oxidase. B) Example SMLM image of CD56 receptors in a fixed HEK293T cell under GLOX condition. C) Localization precision asnalysis yield ≈16 nm resolution in the lateral plane. B,C) Reproduced under the terms of the CC‐BY license.^[^ ^67^ ^]^ Copyright 2020, The Authors. Published by Springer Nature. D) Oxygen scavenging mechanism of photostabilizing buffers based on protocatechuate dioxygenase. E) 3D SMLM image of the microtubules in a fixed Cos7 cell under PCA/PCD buffer supplemented by 2 × 10^−3^ m COT. F) Enhanced photon budget allowed to resolve the tube structure with ≈35 nm diameter of the microtubules in axial dimension. E,F) Reproduced under the terms of the CC‐BY license.^[^ ^68^ ^]^ Copyright 2013, The Authors. Published by PLOS. G,H) Photostabilizing mechanism of photostabilizing buffers based on G) pyranose oxidase, and H) methylene blue. I) Direct comparison of the SMLM images of the microtubules in fixed HeLa cells under GLOX buffer (left) and MB‐thiol buffer (right). Reproduced with permission.^[^ ^69^ ^]^ Copyright 2013, American Chemical Society. J) Multicolor SMLM images of plenctin (blue, Alexa488), keratin (green, Alexa555), and *β*4 integrin (red, Alexa647) in a fixed PA‐JEB/*β*4 cell under OxEA condition. K) OxEA better preserves the number of blinking events per frame over time due to the stable pH. J,K) Reproduced under the terms of the CC‐BY license.^[^ ^71^ ^]^ Copyright 2016, The Authors. Published by PLOS. L) SMLM image of the microtubules in a fixed Cos7 cell under 28‐days old sulfite buffer. M) Long‐term preservation of the fluorophores in the sulfite buffer. L,M) Reproduced with permission.^[^ ^72^ ^]^ Copyright 2018, American Society for Cell Biology.

Glucose oxidase (GLOX) is a highly efficient oxygen scavenger and can reduce the concentration of dissolved oxygen to ≈15 × 10^−6^ m within 3 min.^[^ ^151^ ^]^ GLOX mediates the oxidation of *β*‐D‐glucose by using molecular oxygen as an electron acceptor to produce D‐glucono‐*δ*‐lactone and hydrogen peroxide (H_2_O_2_) (**Figure** **2**A).^[^ ^152^ ^]^ D‐glucono‐*δ*‐lactone is spontaneously hydrolyzed to gluconic acid, which induces the spontaneous acidification of the GLOX‐based imaging buffer.^[^ ^153^ ^]^ H_2_O_2_ promotes the production of ROSs in solution and, thus, leads to photobleaching. A separate enzyme, catalase, is often employed in GLOX system to decompose H_2_O_2_ to water and molecular oxygen.

Photoswitching of organic fluorophores, which is utilized in SMLM, requires an aqueous imaging buffer so that the photoswitching‐inducing reagents can freely diffuse.^[^ ^21^, ^22^, ^66^, ^154^ ^]^ Carbocyanine dyes with long internal chains, such as Cy5, Cy5.5, Cy7, and Alexa647, can be reversibly turned off by the photoaddition reactions of small thiols such as *β*ME, *β*‐mercaptoethylamine (MEA, cysteamine), and L‐cysteine methyl ester (L‐Cys‐ME) (Figure 1A).^[^ ^154^ ^]^ Small phosphines such as tris(2‐carboxyethyl)phosphine (TCEP) also reversibly quench the fluorescence of cyanine dyes by forming adducts.^[^ ^66^ ^]^ Under optimized imaging buffer conditions that containing both GLOX and thiols, Alexa647 (which is a classic best‐performing dye in STORM) provides >5000 photons per switching cycle at an ≈0.001 duty cycle, which corresponds to a spatial resolution of ≈10 nm.^[^ ^23^, ^47^ ^]^ Thus, GLOX‐based imaging buffers that are supplemented with small thiols have been used as a general imaging medium that postpones the irreversible photodamage of fluorophores during imaging by STORM approaches. A recent study combined dSTORM and lattice light‐sheet approaches for 3D volumetric super‐resolution imaging, which enabled the acquisition of the 3D distribution of plasma membrane receptors (i.e., CD56, CD2, and CD45) in whole cells in a GLOX‐based imaging buffer (Figure 2B,C).^[^ ^67^ ^]^

Improved enzymatic oxygen scavenging systems based on protocatechuate‐3,4‐dioxygenase (PCD) were introduced to further reduce the concentration of dissolved oxygen.^[^ ^151^ ^]^ PCD is a well‐characterized, multimeric enzyme that catalyzes the conversion of protocatechuic acid (PCA) to *β*‐carboxy‐*cis*,*cis*‐muconic acid by consuming molecular oxygen (Figure 2D). The PCD system provides a fivefold lower steady‐state O_2_ concentration versus GLOX, supporting ≈140% longer observation times before photobleaching without substantial perturbations of other photophysical characteristics.^[^ ^151^ ^]^ As a triplet quencher, cyclooctatetraene (COT) further stabilizes cyanine dyes,^[^ ^155^ ^]^ and Alexa647 under PCA/PCD + COT conditions support more than 30 000 photons per switching cycle, which provides excellent spatial resolution in 3D STORM imaging (Figure 2E,F).^[^ ^68^ ^]^ Chemical additives (i.e., triplet quenchers or reducing/oxidizing agents) are often employed to obtain better imaging results; however, their use requires careful investigation of the SRM‐specific photophysics, because they may affect various characteristics of the fluorophores such as photon budget, switching kinetics, and duty cycle.

A major disadvantage of GLOX‐ and PCD‐based oxygen scavenging system is the spontaneous pH drop due to the production of carboxylic acids. In solutions with ≈50 × 10^−3^ m of common buffering agents, such as Tris or HEPES, the pH of the imaging buffer that contains GLOX begins to decrease within 1 h, which alters the photophysical properties of organic fluorophores.^[^ ^153^ ^]^ By comparison, PCA/PCD systems better constrain the pH around 7.5–8.0 but such systems also exhibit rapid acidification when the initial pH is near 7.0.^[^ ^151^, ^153^, ^156^ ^]^ An alternative system employs pyranose oxidase (PYOX), instead of GLOX, to maintain anaerobic conditions.^[^ ^156^ ^]^ The final chemical product of PYOX‐based oxygen scavenging systems is a ketone (2‐dehydro‐D‐glucose) (Figure 2G), while solutions with such scavenging systems have constant pH over at least 2 h. As an efficient oxygen scavenger, PYOX supported similar single‐molecule photostabilities for a variety of organic dyes, such as Cy3, Cy5, Alexa647, Atto550, Atto647N, and TAMRA, without notable changes in blinking behaviors. Alternatively, a pH‐stable oxygen scavenger was formulated from the oxygen mediated photoreduction of methylene blue (MB) with appropriate reducing agents such as MEA (Figure 2H).^[^ ^69^ ^]^ This new strategy provided similar oxygen scavenging efficiencies to GLOX enzyme‐based systems without significant acidification of the imaging buffer. This thiol supplemented MB buffer yielded fair performances for the SMLM imaging of microtubules (Figure 2I). In the MB‐based SMLM buffer, the dissolved MB was rapidly discolored and remained dark under oxygen‐depleted conditions. Yet, high concentrations of methylene blue could promote the formation of aggregates during image acquisition and inhibited fluorophore localization around aggregates.

For multicolor SRM, different fluorophores often undergo distinct photoswitching mechanism and therefore each requires specific buffer compositions that limit fluorophore combination. For example, cyanine dyes operate optimally in oxygen‐free conditions but rhodamine dyes do not blink well in the absence of oxygen.^[^ ^47^ ^]^ Oxyrase‐based anaerobic conditions (OxEA) were proposed as global conditions for fluorophores because it supports only small amounts of molecular oxygen.^[^ ^70^, ^71^ ^]^ Oxyrase is a sterile solution of membrane fragments from *Enterococcus coli* that specifically contain enzymes that catalytically convert molecular oxygen.^[^ ^157^ ^]^ OxEA requires DL‐lactate as a substrate and supports 1–2% of the steady‐state molecular oxygen concentration without significantly changing the solution pH, as well as the intracellular functions allowing live‐cell imaging.^[^ ^158^ ^]^As a result, OxEA permits efficient photoswitching of both cyanine and rhodamine fluorophores under the same buffer compositions, enabling simultaneous multicolor GSDIM imaging with Alexa488, Alexa555, and Alexa647 (Figure 2J,K).^[^ ^71^ ^]^

Alternative photostabilizing buffers include the sulfite buffer and the heavy water (D_2_O) instead of normal water (H_2_O).^[^ ^72^, ^73^, ^74^ ^]^ Sodium sulfite is a well‐known oxygen scavenger that has a powerful scavenging efficiency.^[^ ^159^ ^]^ Due to the simple reaction stoichiometry (2Na_2_SO_3_ + O_2_ → 2Na_2_SO_4_), it works well with glycerol at a high concentration without any notable pH perturbation. As a result, a photostabilizing buffer consisting of 80–90% glycerol, MEA, and sodium sulfite provides a high refractive index oxygen‐free imaging condition and a long‐term storage capability at room temperature up to 28 days (Figure 2L,M).^[^ ^72^ ^]^ Replacement of H_2_O with D_2_O with heavy hydrogen atoms affects the hydrogen bonds and the excited‐state proton‐transfer reaction between the water molecules and the fluorophores, which generally increases the quantum yield of fluorophores.^[^ ^73^, ^74^ ^]^ As a result, in D_2_O‐based imaging buffers, oxazine fluorophores such as Atto655 showed two‐times higher quantum yield and photon numbers per switching cycle,^[^ ^73^ ^]^ and cyanine dyes such as Alexa647 showed 1.1‐ to 2.7‐times higher performance depending on the emission spectra.^[^ ^74^ ^]^ D_2_O is also known to increase the photon number of FPs leading better SMLM imaging performance.^[^ ^75^ ^]^

### 2.2 Self‐Healing Dyes

**Figure 3:**
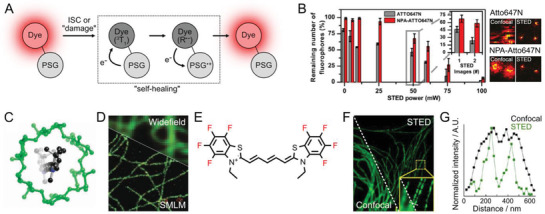
Photostabilizing modifications of organic dyes. A) Schematic diagram of mechanism of self‐healing dyes. A photostabilizing group (PSG) linked to a dye rescues the excited triplet state (^3^T_1_) through the radical state (R˙^−^), by the intramolecular electron transfer, back to the ground state. B) Application of self‐healing dyes to STED imaging. NPA‐linkied Atto647N showed better survival probability (left graph) with lower photoblinking (right images). Reproduced under the terms of the CC‐BY license.^[^ ^76^ ^]^ Copyright 2019, The Authors. Published by IOP Science. C) Schematic illustration for the encapsulation of a cyanine dye by *α*‐cyclodextrin. Reproduced with permission.^[^ ^160^ ^]^ Copyright 2008, The Royal Society of Chemistry. D) 2‐color GSDIM image of PVA‐encapsulated Atto532 (green, microtubules) and Atto565 (red, peroxysomes) dyes in a fixed PtK2 cell. Reproduced with permission. ^[^ ^34^ ^]^ Copyright 2008, Springer Nature. E) Cyanine dye with electron‐withdrawing fluorenes (red). F) STED imaging of the microtubules in a fixed HeLa cell with PhoxBright 430 dye that is internally modified with electron‐removing phosphine oxide. G) Comparison of the line profile between confocal and STED images. F,G) Reproduced with permission.^[^ ^79^ ^]^ Copyright 2017, American Chemical Society.

Self‐healing dyes are synthetic organic fluorophores with covalently attached photostabilizing groups (PSG), such as COT, 4‐nitrobenzyl alcohol (NBA), and 6‐hydroxy‐2,5,7,8‐tetramethylchroman‐2‐caboxylic acid (Trolox) (**Figure** **3**A).^[^ ^161^, ^162^, ^163^, ^164^, ^165^, ^166^ ^]^ In buffer‐based systems, diffusion‐mediated reduction‐oxidation reactions restore two vulnerable states of fluorophores. Different pairs of reductants and oxidants are generally required for efficient photostabilization. But in self‐healing dyes, the PSG plays both roles during the fluorescence recovery, which is mediated by electron transfer reactions (Figure 3A). No negative effects with respect to fluorophore brightness were reported for PSG attachment on fluorophores.^[^ ^161^ ^]^ The direct covalent linkage between the fluorophore and PSG effectively increases the local concentration of the stabilizer around the fluorophores, which potentially leads to fast internal redox reactions. Further careful modification allowed intracellular labeling of self‐healing dyes for live‐cell applications.^[^ ^167^ ^]^ However, biological applications of self‐healing dyes are currently limited, owing to the poor understanding of the influence of the biological environment on the self‐healing process and to their cost‐inefficient synthesis.^[^ ^166^ ^]^

Self‐healing dyes have advantages in SRM applications in terms of brightness and photostability (Figure 3B).^[^ ^76^, ^165^ ^]^ Nitrophenylalanine (NPA)‐conjugated Atto647N dye and nitrophenylacetic acid‐conjugated Star635P were prepared for STED imaging. Substantially larger fractions of self‐healing dye molecules survived after STED imaging, and their average brightness was enhanced. Likewise, Cy5 was separately conjugated to NPA, COT, and Trolox and the photoswitching behaviors were investigated in the presence of TCEP or MEA as switching agents. The photostabilizer largely influenced the photoswitching kinetics, and the concentration of TCEP could be tuned to optimize either the reactivation percentage or the fluorescence on times. Yet the use of MEA to induce the photoswitching caused a significant reduction in the average photobleaching lifetime, while the reactivation percentage remained constant. These indicate that intramolecular photostabilization for SMLM requires careful attention to various photophysical characteristics.

### 2.3 Encapsulation of Fluorophores

Fluorophore encapsulation within a host can obstruct the access of molecular oxygen to the fluorophore and is an alternative way to enhance photostability (Figure 3C). Fluorophores encapsulated within a hydrophobic environment of host molecules are prevented from interactions and reactions with water molecules. Encapsulation of Rhodamine 6G fluorophores into cucurbit[7]uril supramolecular hosts greatly improved the photostability of Rhodamine 6G, and the resulting complex could be applied to single‐molecule detection.^[^ ^168^ ^]^ Encapsulation of Cy5 in cyclodextrin hosts also improves fluorophore photostability.^[^ ^160^ ^]^ Photobleaching of a hydrophobic borondipyrromethene (BODIPY) derivative was suppressed by linking it with hydrophilic polyglycerol dendrimers that served defensive moieties.^[^ ^169^ ^]^

Highly encapsulated environment quarantines the fluorophore from dissolved oxygen, thus prolongs triplet lifetime and reduces photobleaching. The lifespans of the triplet states of organic fluorophores, which are typically on the order of a few microseconds, can be substantially extended by integrating fluorophores into poly‐(vinyl alcohol) matrix (PVA). The poor oxygen permeability into PVA decreases triplet‐state quenching by oxygen. Approximately 95% of Cy5 molecules in a PVA matrix can transit to an off state at a given time without the addition of extra switching agents. This enabled SMLM imaging of Cy5‐labeled microtubules in PVA with no chemical additives.^[^ ^77^ ^]^ PVA‐assisted photoblinking was successfully employed for nonblinking fluorophores such as Atto532 and Atto545 for SMLM by protecting the fluorophores from dissolved oxygen species to maintain sufficient triplet state lifetime for photoblinking (Figure 3D).^[^ ^34^ ^]^

### 2.4 Structural Modifications of Organic Dyes

The incorporation of electron‐withdrawing groups (EWGs) into the fluorophore structure is a general and established method to reduce the reactivity of organic molecules with ^1^O_2_ and other ROS species, which ultimately improves photostability. The introduction of EWGs into molecules reduces the levels of the lowest unoccupied molecular orbital (LUMO). The energy level of the triplet state is also reduced, which suppresses the reactivity of the molecule to both molecular oxygen and singlet oxygen.

This tactic has been employed to generate photostable BODIPY, cyanine, and xanthene fluorophores (Figure 3E).^[^ ^170^, ^171^, ^172^, ^173^, ^174^, ^175^ ^]^ A threefold improvement in photostability with a modest 40% reduction in fluorescence brightness was achieved by perfluorination of aromatic rings in cyanine dyes. Moreover, the modification of a merocyanine dye with a cyano group produced a new fluorophore with a significantly reduced ^1^O_2_ response, resulting in an impressive 40‐fold reduction in photobleaching.^[^ ^171^ ^]^ Appending rhodamines and cyanine dyes with sulfonate groups, which are EWGs, can also lead to improved photostability and enhanced solubility.^[^ ^173^, ^176^, ^177^ ^]^ The recently reported PhoxBright 430 fluorophore contains electron‐removing phosphine oxide and has excellent photostability that permits long‐term STED imaging (Figure 3F,G).^[^ ^79^ ^]^ The introduction of an CH_2_CF_3_ moiety at either the nitrogen or fluorine atoms in rhodamines at positions 2 and 7 of xanthene slow radical formation and photobleaching. These fluorophores are cell‐permeable and have been successfully applied to image tubulin filaments in live cells via STED.^[^ ^79^ ^]^

Silicon‐substituted rhodamine (SiR) is a near‐infrared (NIR) fluorophore that recently generated various live‐cell probes due to cell permeability and the benefits of NIR (i.e., deep penetration, minimal background and photodamage).^[^ ^178^, ^179^, ^180^ ^]^ Fluorinated SiR offered high photostability and high quantum yield in water for red‐shifted STED imaging with depletion laser at 800 nm.^[^ ^78^ ^]^ Replacement of the *N*,*N*‐dimethylamino groups on the classic rhodamine backbone with *tert*‐butylamino groups yields a new class of rhodamine‐type fluorophores that have excellent photostability and high brightness for live‐cell STED imaging.^[^ ^80^ ^]^ Moreover, the introduction of two methoxy groups on benzene moiety of SiR family improves brightness by 20‐fold, as well as the photostability, due to steric effects that may hinder any perturbations on the xanthene moiety, resolving mitochondrial cristae in living cells by STED microscopy.^[^ ^81^ ^]^

### 2.5 Structural Modification of Fluorescent Proteins

Modifying the protein structure near the chromophore is a key strategy to increase the photostability of FPs.^[^ ^63^ ^]^ The rational design of such modifications relies on understanding the mechanisms of photo‐destruction of the chromophore. The replacement of an amino acid residue by a bulkier one in the chromophore environment can provide relatively higher stability against photobleaching.^[^ ^59^ ^]^ It is likely that the effects of such modifications originate from the suppression of the *cis*‐*trans*‐isomerization and protonation/deprotonation of the chromophore group, as well as improved isolation of the chromophore from oxygen molecules. In the GFP family, photobleaching is involved with photoinduced electron transfer via an electron hopping mechanism through Tyr145.^[^ ^181^ ^]^ Substitution of Tyr145 by less effective election‐accepting residues resulted in protein mutants with much higher photostabilities.^[^ ^181^ ^]^ SiriusGFP is the best GFP variant with respect to photostability and contains two key mutations, which are S147R and S205V, that significantly improve photostability versus other GFPs.^[^ ^82^ ^]^ The strong resistance of SiriusGFP to photobleaching permitted robust 3D SIM imaging with high‐quality 3D reconstruction.^[^ ^82^ ^]^ However, the mutation also decreased the quantum yield three fold lower than other GFPs. Therefore, when engineering FP for photobleaching, it is desirable to optimize both brightness and photobleaching at the same time.^[^ ^182^ ^]^

### 2.6 Cryogenic Super‐Resolution Fluorescence Microscopy

At cryogenic temperatures, the diffusion of reactive oxygen species including molecular oxygen is arrested, therefore the oxygen‐mediated photobleaching pathways become extremely inefficient. Consequently, the lifetime of the triplet state can increase by several orders of magnitude.^[^ ^183^ ^]^ In addition, cryofluorescence microscopy can directly image cryo‐immobilized biological specimen using rapid freezing methods (i.e., vitrification) that preserves biological structures in a near‐native state.^[^ ^184^ ^]^ Also, correlative light and electron microscopy (CLEM) can combine the powers of the two microscopies: fluorescence microscopy offers molecular specificity while electron microscopy (EM) provides atomic resolution and cellular context.^[^ ^185^, ^186^ ^]^

Despite these unique benefits, cryo‐SRM faces many technical hurdles. First, long‐working distance objective lenses used in cryogenic microscopy degrades the diffraction‐limited resolution.^[^ ^187^ ^]^ Long working distance objectives prevent cryogenic sample from contacting the optical system in ambient temperature. The numerical apertures (NA) of typical objectives in cryofluorescence is 0.7–0.9, whereas NA of immersion lenses in ambient SRM is 1.2–1.5.^[^ ^188^ ^]^ Second, high excitation intensity can lead into local devitrification and permanent transition to crystalline state. Since ≈MW/cm^2^ are required for efficient depletion for STED and ≈kW/cm^2^ are required for blinking‐based SMLM, these methods can induce local heating of vitrified samples.^[^ ^188^ ^]^ Third, cryogenic temperatures significantly slow down many photophysical pathways including switching reactions required for achieving diffraction‐unlimited resolution. In cryo‐STED, the lifetimes of singlet‐excited states also become much longer, requiring excessive depletion intensity that can induce sample heating.^[^ ^83^ ^]^ For SMLM of dyes with switching agents in solution, the diffusion of switching agent is arrested at cryogenic temperatures and cannot support switching reactions.

Due to the above‐mentioned challenges, there have been few demonstrations of cryo‐SRM to date. ^[^ ^188^ ^]^ Purely optical techniques such as SIM have been demonstrated at cryogenic temperatures, albeit the resolution is inherently lower than that in ambient SIM due to the objective NA.^[^ ^89^, ^90^ ^]^ Cryo‐STED is challenged by the higher depletion power for depleting excited molecules with longer lifetimes at cryogenic temperature.^[^ ^83^ ^]^ Since SOFI does not rely on high laser intensity for efficient off‐switching, cryo‐SOFI with three‐fold resolution improvement was demonstrated in conventional cryofluorescence microscope.^[^ ^88^ ^]^ Cryo‐SMLM is largely limited to fluorescent proteins that do not require diffusing switching agents.^[^ ^84^, ^87^, ^89^ ^]^ However, due to the low photon outputs of FPs, the resultant SMLM resolution was not any better than ambient SRM with organic dyes even when using a high NA lens.^[^ ^85^ ^]^ Self‐blinking dyes such as Atto647N was used to localize multiple sites within a molecule to quantitate the conformational states of proteins.^[^ ^86^ ^]^

The compatibility to vitrified samples make cryo‐SRM a powerful tool for CLEM.^[^ ^188^ ^]^ By avoiding chemical fixation and preserving cellular structures in near native state, cryo‐SIM and cryo‐SMLM combined with electron tomography or block‐face EM unveiled new cellular ultrastructures.^[^ ^89^, ^189^ ^]^ The advancements in microscope lens and bodies specialized in cryo‐SRM as well as fluorophores with reliable switching kinetics in cryogenic temperatures will leverage cryo‐SRM to be a new tool of choice for in situ structural biology.

### 2.7 Nanoparticles in Super‐Resolution Microscopy

The rapid growth of nanomaterial science and engineering has yielded a new class of fluorophore that addresses several disadvantages of organic dyes and FPs.^[^ ^191^ ^]^ Fluorescent nanoparticles have distinct structures and photophysical characteristics than conventional fluorophores, offering excellent brightness and photostability. The rational design of these nanoscopic particles permits further modifications of the photophysical properties, including photobleaching resistance, and makes them promising fluorescent probes for SRM.^[^ ^41^, ^191^, ^192^ ^]^

**Figure 4:**
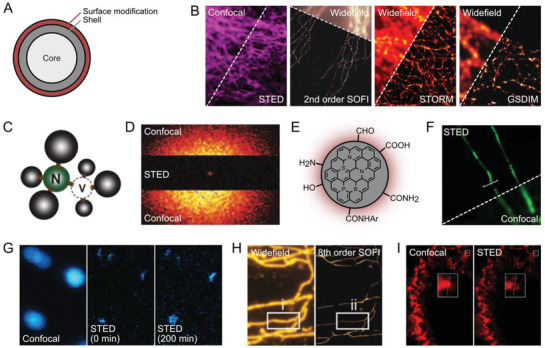
Photostable nanoparticles in super‐resolution microscopy. (A) A typical core‐shell structure of QDots. The protective shell structure enhances the photostability. B) Super‐resolution applications with commercially available QDots (STED: QD705, microtubules in a fixed HeLa cell; SOFI: QD625, microtubules in a fixed 3T3 cell; STORM: QD565, microtubules in a fixed GepG2 cell; GSDIM: microtubules in a fixed PtK2 cell). Reproduced with permission.^[^ ^42^, ^91^, ^93^, ^94^ ^]^ STED: Reproduced with permission.^[^ ^94^ ^]^ Copyright 2016, American Chemical Society. SOFI: Reproduced with permission.^[^ ^42^ ^]^ Copyright 2009, PNAS. STORM: Reproduced with permission.^[^ ^93^ ^]^ Copyright 2015, American Chemical Society. GSDIM: Reproduced with permission.^[^ ^91^ ^]^ Copyright 2011, American Chemical Society. QDots have been used in multiple techniques due to their high photostability and characteristic photophysics. C) NV^−^ color center in tetrahedral carbon array of diamond. Reproduced under the terms of the CC‐BY license.^[^ ^190^ ^]^ Copyright 2012, Optica Publishing Group. D) STED imaging of NV^−^ center in a bulk diamond with extreme depletion intensity, yielding 8 nm of spatial resolution. Reproduced with permission.^[^ ^97^ ^]^ Copyright 2009, Springer. E) Schematic of structure of fluorescent CDots. F) Example SMLM application of a nitrogen‐doped CDot in the presence of methyl viologen (tunneling nanotubules in a live 4T1 cell). Reproduced with permission.^[^ ^103^ ^]^ Copyright 2019, Springer Nature. G) Bleaching‐resistant STED imaging on UCNP for 200 min under continuous scanning. Reproduced with permission.^[^ ^104^ ^]^ Copyright 2017, Springer Nature. H) Eighth‐order SOFI imaging on continuously blinking PDots (microtubules in a fixed BS‐C1 cell). Reproduced with permission.^[^ ^108^ ^]^ Copyright 2020, The Royal Society of Chemistry. I) STED imaging of mitochondria by TPA‐T‐CyP AIE nanoparticle (mitochondria in a live HeLa cell). Reproduced with permission.^[^ ^110^ ^]^ Copyright 2018, Springer Nature.

Quantum dots (QDots) are the first generation of nanoparticle fluorophores with energy gaps that are modulated by quantum confinement effects, which arise from the small sizes (≈10 nm) of the QDots (**Figure** **4**A).^[^ ^193^, ^194^ ^]^ As a result, the size of QDots tunes the energy of the emitted fluorescence, and the resulting fluorescence exhibits a narrow emission band, which facilitates multicolor imaging with super‐resolution techniques.^[^ ^64^ ^]^ Generally, QDots have large absorption cross‐sections and high quantum yields that support greater brightness than organic fluorophores or FPs. QDots with core‐shell structures also have improved photostabilities versus solid QDots. Now QDots with a variety of core‐shell structured are commercially available and broadly used for STED, SIM, and other super‐resolution applications (Figure 4B).^[^ ^42^, ^91^, ^92^, ^93^, ^94^ ^]^ A recent application of PAINT imaging demonstrated that the superior brightness of QDots supported enhanced resolution versus organic fluorophores.^[^ ^95^ ^]^

Fluorescent nanodiamonds (FNDs) are fine powders of diamond that contain a chromophore, which comprises a nitrogen atom with a nearby negatively‐charged vacancy (NV^−^ center) (Figure 4C).^[^ ^65^, ^195^ ^]^ NV^−^ center has a broad emission spectrum that is suitable for STED microscopy while also exhibiting a light‐controllable dark state with a long lifetime that permits RESOLFT microscopy.^[^ ^97^, ^98^ ^]^ NV^−^ center are also found in a small‐sized diamond grains, fluorescent nanodiamond (FND), which can be used as a fluorescent probe for SRM.^[^ ^196^ ^]^ In terms of fluorescence imaging, one of the most notable photophysical properties of NV^−^ center is extreme robustness against photobleaching. The notable photostability of NV^−^ center allows extremely high depletion intensity for exceptionally high resolution enhancement in STED‐type strategies, that resulted in resolutions of ≈10 nm for STED, GSD, and RESOLFT approaches (Figure 4D).^[^ ^96^, ^97^, ^98^, ^99^ ^]^

Carbon dots (CDots) are fluorescent carbon‐based nanoparticles that are 2–5 nm in diameter and are biocompatible, water‐soluble, and can be cost‐effectively produced on large scales (Figure 4E).^[^ ^197^ ^]^ Various functional groups such as −OH, −NH_2_, and −COOH on the surface of CDots can be easily conjugated to biomolecules.^[^ ^198^ ^]^ While the mechanism of fluorescence in CDots remains debated,^[^ ^199^ ^]^ CDots provide highly stable fluorescence signals without notable photoblinking that supports efficient STED imaging (Figure 4F).^[^ ^100^, ^103^ ^]^ Appending an electron acceptor molecule to CDots further modulates photophysical properties, including light‐sensitive fluorescence switching of CDots that are beneficial for SMLM.^[^ ^101^, ^102^ ^]^

Highly‐doped upconversion nanoparticles (UCNPs) that have low saturation intensities are attractive for STED microscopy (Figure 4G).^[^ ^104^, ^105^, ^106^, ^200^ ^]^ Small polymer dots (PDots) exhibit spontaneous fluctuations in fluorescence that make them suited for fluctuation‐based super‐resolution techniques, such as super‐resolution optical fluctuation imaging (SOFI) (Figure 4H).^[^ ^107^, ^108^ ^]^ Several aggregation‐induced emission (AIE) nanoparticles have large STED cross‐sections and strong resistance to photobleaching. As a result, such AIE nanoparticles are excellent candidates for long‐term STED imaging (Figure 4I).^[^ ^109^, ^110^, ^111^, ^201^ ^]^

These nanoparticles still have several disadvantages, such as their relatively large sizes versus organic dyes, the requirement for surface modification for labeling, and biocompatibility. Despite such disadvantages, nanoparticles are rapidly emerging as promising probes for SRM due to their strong resistance to bleaching.

## 3 Nonfluorescent States As Safeguards for Protecting from Photobleaching

The previous discussion introduced conventional methods to increase the photostabilities of fluorophores and how these strategies were applied to super‐resolution imaging. Chemical additives and other variables can perturb the live‐cell physiology.^[^ ^202^ ^]^ Moreover, it can be difficult to establish optimal conditions for all fluorophores in applications that employ multiple types of fluorophores for multicolor SMLM to investigate either live or fixed cells. As super‐resolution imaging uses on–off transitions for overcoming the optical diffraction limit (Chapter 1.2), it is possible to actively use off‐states or prepare alternative dark states to protect fluorophores from photobleaching. Depending on the fluorophores and the imaging conditions, some remaining populations can be photobleached before they transit to a dark state. However, general off‐switching rate is much faster than the photobleaching rate in this strategy so most of dyes can avoid photobleaching in the nonfluorescent state.

Safeguarding dark states for SRM can be prepared in various ways. Spontaneous blinking chemistry can bypass the requirement for intense laser irradiation and chemical additives for photo‐induced blinking. Moreover, utilizing different switching rates in various local environments can be important for maintaining the fluorophore in the dark state. Also, spectrally distinct dimers formed from monomer reservoirs can improve the photostability of certain fluorophores. Another emerging tactic to extend imaging time is to provide additional energetic pathways, in lieu of photobleaching, by using Förster resonance energy transfer (FRET). All of these strategies require more interrogation and thus are further discussed in the following text.

### 3.1 Protected STED Nanoscopy by the Use of Photoswitchable Probes

**Figure 5:**
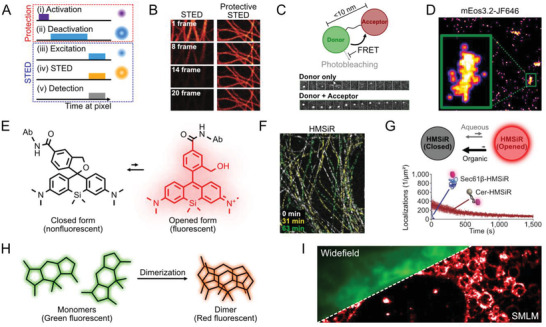
Safeguarding strategies using dark states as shelter from photobleaching. A) Illumination sequence in Protected STED. Fluorophores around the focal spot evacuate to a dark state from photobleaching induced by intense STED light. B) Conventional versus Protected STED images. Protected STED provides much extended observation time (keratin in a live HeLa cell). Reproduced with permission.^[^ ^112^ ^]^ Copyright 2016, Springer Nature. C) The concept of FRET‐enhanced photostability using a photo‐modulated donor fused to a photostable acceptor. FRET efficiently suppresses photobleaching of donor molecule. Reproduced under the terms of the CC‐BY license.^[^ ^113^ ^]^ Copyright 2018, The Authors. Published by Springer Nature. D) SMLM on CENP‐A with FRET‐modulated mEos3.2 in a live mouse embryonic stem cell. Reproduced under the terms of the CC‐BY license.^[^ ^113^ ^]^ Copyright 2018, The Authors. Published by Springer Nature. E) Spontaneous blinking reaction of HMSiR. F) SMLM of HMSiR allowing investigation on structural dynamics more than 1 h (microtubules in a live Vero cell). Reproduced with permission.^[^ ^38^ ^]^ Copyright 2014, Springer Nature. G) On–off equilibria of HMSiR in aqueous and organic environments (top). The prolonged dark state in lipid results in notably longer acquisition of SMLM (bottom). Reproduced with permission.^[^ ^116^ ^]^ Copyright 2017, Springer Nature. H) Transient red‐shifted ground state dimer (D_II_) of BODIPY dyes. I) SMLM of lysosomes by D_II_ formation of a BODIPY analog, LysoTracker Green (lipid droplets in a live U2OS cell). Reproduced under the terms of the CC‐BY license.^[^ ^119^ ^]^ Copyright 2017, The Authors. Published by Springer Nature.

The coupling of photoswitchable FPs with STED nanoscopy can prevent photobleaching by both excitation and depletion irradiation. Photoswitchable FPs can be driven into two distinct off states in this system, namely a deactivated state and an activated form in the ground state. The deactivated states of FPs can be protected from bleaching by the STED and excitation lasers. The STED laser then triggers the active form of the ground state, which subsequently acts on the excited state of the active protein. This strategy is known as protected STED, which is a technique that employs a series of light pulses sequentially applied to individual designed subregions across an imaging area (**Figure** **5**A). Initially, all of the photoswitchable fluorescent proteins in a diffraction‐limited area are turned on. Then, a donut‐shaped beam deactivates the fluorophores along the edge of focal spot. Finally, the active fluorophores that remain in the center of the focus are excited by a laser surrounded by a second donut that is created by the STED laser. As a reduced number of fluorescent molecules absorb excitation and STED light at any given time, the protected STED is more likely to result in less photodamage to fluorophores over prolonged imaging durations (Figure 5B). Moreover, the near‐complete suppression of fluorescence outside the target area provided by protected STED increases the contrast of this technique and ultimately provides greater resolution than the conventional STED.^[^ ^112^ ^]^

### 3.2 FRET Enhanced Photostability

The FRET pathway competes with standard photobleaching pathways to increase the photostability of donor fluorophores.^[^ ^203^ ^]^ This is achieved by the presence of single‐molecule FRET pairs that consist of a photomodulatable (PM) donor fluorophore in molecular proximity (<10 nm) to a photostable acceptor dye.^[^ ^113^ ^]^ A photostable organic dye like JF646 that is in close spatial proximity to a PM donor fluorophore, such as mEos3.2 or PA‐JF549, can result in FRET, in which non‐radiative energy transfer occurs through dipole‐dipole coupling between the PM and acceptor dye. FRET provides additional energetic pathways that are alternative to photobleaching and therefore alter the excited‐state kinetic and photophysical properties, such as the lifetime of fluorescence and photostability of the PM donor (Figure 5C). This approach enables single‐molecule tracking of CENP‐A at centromeres in live mammalian cells for extended periods (Figure 5D).^[^ ^113^ ^]^

### 3.3 Spontaneously Blinking Fluorophores

Spontaneously blinking fluorophores undergo on–off transitions with characteristics that are independent of the laser irradiation strength or thiol concentration.^[^ ^38^, ^204^, ^205^ ^]^ The off‐state can be engineered to be extremely stable, thus reducing the chance for the system to proceed along a photobleaching pathway. Rhodamine derivatives appended with a nucleophilic group can exist in a thermal equilibrium between a fluorescent “open” form and a nonfluorescent “spirocyclic” form, both in the ground state.^[^ ^178^, ^206^ ^]^ This phenomenon can be regulated by the nucleophilicity of the intramolecular nucleophile and/or the electrophilicity of the fluorophore. The ratio of fluorescent open to nonfluorescent spirocyclic forms, as well as the duration of the on‐transition state, are key parameters for regulating the blinking phenomenon and can be controlled.^[^ ^38^ ^]^ HMSiR is a SiR with a hydroxy group and was the first fluorophore to achieve spontaneous blinking with suitable kinetics and photophysics for SMLM (Figure 5E). HMSiR blinks properly in the absence of additives and without exposure to extreme illumination. HMSiR was used in combination with a spinning‐disk confocal microscope to successfully image nuclear pore structures that were located far above the coverslip and for time‐lapse imaging of microtubules for one hour in live cells (Figure 5F). The HEtetTFER fluorophore is a derivative of Rhodamine B that emits green light and undergoes spontaneous blinking. While this fluorophore has yet to be used for live‐cell applications, it has been applied for fixed cell SMLM.^[^ ^115^ ^]^ As HMSiR emits fluorescence in the NIR region and HEtetTFER emit green light, the two spontaneously blinking fluorophores are easily resolved spectrally and thus serve as a useful fluorophore combination for dual‐color SMLM.^[^ ^115^ ^]^

The spontaneous blinking equilibrium of HMSiR is sensitive to the environment.^[^ ^116^ ^]^ The dark state of HMSiR is much more stable in a lipid environment than in aqueous media (Figure 5G). Thus, HMSiR molecules embedded in hydrophobic membranes can serve as a stable reservoir for dark state in which the photobleaching pathways are out of reach. Lipophilic probes made from HMSiR, referred to as high‐density environmentally sensitive (HIDE) probes, supported notably prolonged acquisition of SMLM movies of membranous organelles in living cells.^[^ ^116^ ^]^

HIDE probes for long‐term STED imaging have been generated from rhodamine dyes with carboxylic groups that can spontaneously form nonfluorescent spirocylic form.^[^ ^207^ ^]^ For instance, two‐component HIDE probes can be generated from clickable SiR dye and high‐density lipid probes with biorthogonal group.^[^ ^207^ ^]^ Ceramide lipid with *trans*‐cyclooctene (TCO) moiety allowed for labeling of Golgi apparatus with tetrazine‐tagged SiR (SiR‐Tz) for live‐cell STED imaging.^[^ ^114^ ^]^ The combined use of a plasma membrane tetrazine ligation probe, DiI‐TCO, and SiR‐Tz enabled the visualization of filopodia dynamics over 25 min by using live‐cell STED nanoscopy.^[^ ^117^ ^]^ The tetrazine moiety has dual functionalities in that they are biorthogonal while also being fluorogenic. Fluorescence is selectively restored when tetrazine moieties convert to dihydropyridazine upon interaction with the target probe. Therefore, high‐density labeling can be achieved without notable non‐specific staining.^[^ ^208^ ^]^ For two‐color HIDE‐STED, strain‐promoted azide‐alkyne cycloaddition reaction (SPAAC) between an azide and a dibenzoazacylooctyne (DBCO) was used as an orthogonal reaction to tetrazine ligation. The SPAAC pair of DiI‐N_3_ and SiR‐DBCO supported similarly long acquisition of STED images to the combination of DiI‐TCO and SiR‐Tz.^[^ ^118^ ^]^ For a spectrally discernable HIDE probe, tetrazine fluorazetidine carborhodamine was synthesized, named as Yale595 and coupled to TCO‐containing lipid probes.^[^ ^118^ ^]^ Two‐component HIDE probes enable facile long time‐lapse imaging and the versatility to label a variety of cellular targets, as this strategy does not require transfection, cell permeabilization, oxygen depletion, or pre‐bleaching.

### 3.4 Dimerized Dyes from a Lasting Source of Monomer

BODIPY dyes are able to form at least two distinct ground state dimers, dimer I (D_I_) and dimer II (D_II_).^[^ ^209^ ^]^ While D_I_ is not fluorescent, D_II_ has a close absorption spectrum to the monomer and emits red‐shifted fluorescence.^[^ ^210^ ^]^ A recent work has revealed D_II_ exhibits yellow excitation and red emission that can be easily distinguished from the monomer by a spectrally distinct pair of laser and emission filter (Figure 5H).^[^ ^119^ ^]^ The low density of D_II_ allows single‐molecule detection of D_II_ molecules and subsequent SMLM imaging with a high photon budget (Figure 5H,I). Abundant BODIPY monomers, which can be considered as a dark state under D_II_ imaging conditions, serve as an infinite source of building blocks for D_II_. The resulting lifetime of BODIPY D_II_ far exceeds the lifetime of HIDE probes, showing advantages in the long‐term live‐cell SMLM.^[^ ^116^ ^]^ Conventional BODIPY conjugates provide a simple and versatile strategy to image lysosomes and other sub‐cellular structures such as lipid droplets to be imaged at the nanoscale in live mammalian cells (Figure 5I).^[^ ^119^ ^]^

### 3.5 Chemical Caging of Fluorescent Proteins

mCherry was found to undergo photoswitching in the presence of *β*ME as reducing agent and utilized for SMLM.^[^ ^120^ ^]^ *β*ME can transform mCherry to a blue fluorescent state via two distinctive mechanisms; the reduction of the chromophore or covalently adding to the C_*β*_ of the chromophore's tyrosine. Up to 80% of the fluorescent state of mCherry can be retrieved from a chemically induced dark state via violet light illumination or by washout. Surprisingly, the number of photons per mCherry blinking events recorded after washout was 54% higher than those photoactivated in the presence of *β*ME.^[^ ^121^ ^]^ The chemical‐caging approach might improve the photostability of mCherry SMLM by decreasing photodamage caused by the activation laser.

## 4 Replacing Bleached Probes with New Ones

**Figure 6:**
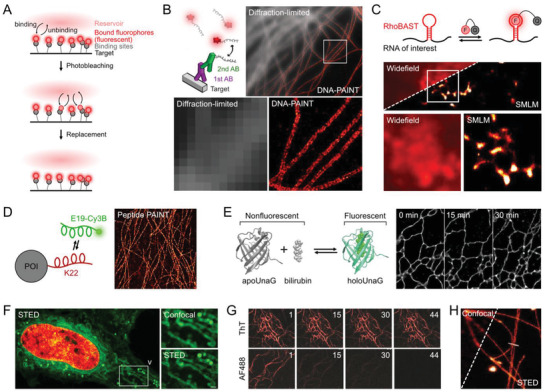
Exchangeable probes applied in super‐resolution microscopy. A) A Scheme for exchangeable probes. Photobleached molecules are continuously changed to fresh molecules. B) A Schematic illustration for DNA‐PAINT and representative DNA‐PAINT image on microtubule in a fixed mammalian cell. Reproduced with permission.^[^ ^211^ ^]^ Copyright 2017, Springer. C) RNA PAINT approach that utilizes RNA aptamer for visualization of mRNA (*FRM1*‐*GFP* mRNA in a live HeLa cell). Reproduced with permission.^[^ ^139^ ^]^ Copyright 2021, Springer. D) Peptide PAINT using a pair of short peptide fragments (K22 and E19) undergoing transient association (microtubules in a fixed U2OS cell). Reproduced with permission.^[^ ^141^ ^]^ Copyright 2020, American Chemical Society. E) Fluorogen‐inducible fluorescent protein as a probe for SMLM. Reversible binding of bilirubin on UnaG protein enables ≈30‐min observation on Sec61*β* on the endoplasmic recticulum (ER) in a live Cos7 cell. Reproduced under the terms of the CC‐BY license.^[^ ^146^ ^]^ Copyright 2020, The Authors. Published by Springer Nature. F) 2‐color STED imaging with exchangeable probes, Nile red (green) and JF646‐labeled Hoechst (red) in a fixed HeLa cell. Reproduced with permission.^[^ ^125^ ^]^ Copyright 2019, American Chemical Society. G) Long‐term STED imaging on amyloid fibril with an exchangeable and binding‐activatable dye, ThT. Reproduced with permission.^[^ ^126^ ^]^ Copyright 2020, The Royal Society of Chemistry. H) STED imaging on *λ*‐DNA stained by YOYO dye. Reproduced under the terms of the CC‐BY license.^[^ ^123^ ^]^ Copyright 2011, Wiley‐VCH.

Fluorophores that transiently attach to cellular structures can renew photobleached labels from surrounding buffer that serves as a reservoir of fresh fluorophores. Exchangeable probes were first introduced for imaging in nanoscale topography (PAINT) as a method for SMLM.^[^ ^26^ ^]^ PAINT does not rely on light to switch fluorescent molecules between on‐ and off‐states. Instead, the same switching or blinking effect is achieved by transient binding.^[^ ^26^ ^]^ In PAINT‐based approaches, interactions between the biomolecular target and fluorescent probe are transient, and the bound but bleached fluorescent molecules are continuously replaced with unbleached and unbound molecules. The key advantage of PAINT‐based techniques is that they bypass the issue of fluorescent probes becoming photobleached over time, which is an unavoidable result of laser excitation (**Figure** **6**A). Unlike other SMLM techniques that are restricted by photobleaching of fluorophores, PAINT‐based approaches rely on the continuous replacement of active fluorescent probes and achieve long imaging times. So long as probes can disperse and arrive at their target molecules, PAINT is easy to implement and does not require specific experimental conditions for photoswitching. Data acquisition can thus proceed beyond the bleaching time scale to enable extended data accumulation and enable the acquisition of higher resolution images.^[^ ^211^ ^]^ Exchangeable probes also help circumvent photobleaching in STED.^[^ ^125^ ^]^

### 4.1 Surface PAINT

PAINT was first introduced by Alexey Sharonov and Robin M. Hochstrasser in 2006.^[^ ^26^ ^]^ This study employed Nile red, whose fluorescence is weak in water and much brighter in lipids, to image large unilamellar vesicles (LUVs). The transient binding events of Nile red molecules to LUVs produced short flashes, which were similar to blinking, to provide super‐resolution data on vesicle positions and diameters over time.^[^ ^26^ ^]^ The original implementation of PAINT, however, was not easily generalized to a broader range of biomolecules because it exploited nonspecific hydrophobic or electrostatic coupling interactions. Membrane receptors were investigated by using universal PAINT, or uPAINT, with nonblinking fluorophores that were conjugated to a ligand or antibody.^[^ ^212^ ^]^ The uPAINT method is based on the continuous and stochastic labeling of membrane biomolecules with fluorescent ligands dispersed in cell exterior during imaging of the samples via oblique illumination.^[^ ^212^ ^]^ A photoactivatable rhodamine fluorophore was coupled to polyethylene glycol to generate an interface‐binding probe to visualize solid‐liquid, liquid‐liquid and liquid‐air interfaces by continuous noncovalent labeling during imaging.^[^ ^213^ ^]^ Continuous labeling is induced by the ongoing exchange among surface‐bound and freely diffusing photo‐activatable and end‐functionalized polymer chains. This approach expands that capability of PAINT to applications in colloids and interfaces, food science, and nanotechnology.^[^ ^213^ ^]^

### 4.2 DNA‐PAINT

The binding avidities of nucleic acids enable programmable target‐probe interactions for PAINT.^[^ ^214^ ^]^ In DNA‐based PAINT (DNA‐PAINT), a single‐stranded DNA molecule is conjugated to a fluorophore (imager).^[^ ^127^ ^]^ This conjugate freely diffuses in the sample and recognizes complementary DNA strands (docking strand) that are intentionally linked to a fixed target of interest (Figure 6B).^[^ ^127^, ^211^ ^]^ DNA‐PAINT leverages transient DNA binding to achieve the same switching or blinking effect like other SMLM methods. Generally, the imager strands in solution appear undetectable by the camera because the molecules diffuse over multiple pixels during the span of a single frame. By comparison, imager strands bound to complementary strands associated with the target molecule become fixed for an extended period of time, producing well‐defined, localized, and detectable bright light. The timescale of binding depends solely on the stability of the DNA duplex, and thus the off‐switching rates are programmable.^[^ ^211^ ^]^ The frequency of binding events, on the other hand, can be modulated by the rate of influx of the imager strands, e.g. by modulating the concentration of the imager strands in the buffer, the number of the docking strands per target or the association constant.^[^ ^135^, ^136^, ^211^, ^215^ ^]^ Consequently, blinking kinetics can be fine‐tuned independently of the dye properties or illumination parameters. The versatile toolbox for in vitro DNA technology can be used to develop fluorescence in situ hybridization (FISH) probe libraries for OligoDNA‐PAINT, super‐resolution writing with photoreactive nucleosides by Action‐PAINT, or labeling of cellular structures by antibody, aptamer, affimer, click chemistry or genetic incorporation.^[^ ^130^, ^131^, ^132^, ^133^, ^134^ ^]^ Moreover, the use of DNA‐based imaging probes permits multiplexing via the use of sequential hybridization, which is limited only by the number of orthogonal DNA sequences.^[^ ^128^ ^]^ Super‐resolution imaging with nine targets was demonstrated by repeated imaging, washing, and reintroduction of new imager strands.^[^ ^128^ ^]^ The predictability and tunability of DNA binding and unbinding events, combined with bleaching resistance, allow for the precise and quantitative analysis of counting single molecules, which is a technique called quantitative PAINT (qPAINT).^[^ ^129^ ^]^ The optimization of experimental conditions and application of complex drift correction techniques allowed true molecular‐scale resolution.^[^ ^216^ ^]^

DNA‐PAINT provides many advantages over conventional super‐resolution techniques, but also has limitations. One drawback is that the imager strands are non‐fluorogenic, which has two consequences. First, DNA‐PAINT is limited to optical sectioning techniques such as total internal reflection (TIR) or confocal detection for practical imaging speed.^[^ ^211^, ^217^ ^]^ Secondly, the concentration of non‐fluorogenic imager strands also set an upper limit on the achievable image acquisition speed.^[^ ^135^, ^136^, ^211^, ^215^ ^]^ To increase the speed above the concentration limit, it requires additional means such as preloading of imaging strand or pretreatment of dyes to a photoactivatable dark state.^[^ ^137^, ^138^ ^]^ Moreover, current DNA‐PAINT applications are limited to fixed specimens, and live‐cell imaging may be challenging because of the difficulty of infusing dye‐labeled nucleic acid strands into living cells.^[^ ^211^ ^]^

### 4.3 RNA PAINT

Single‐stranded RNA can form folded structures that enable these molecules to bind to small molecules. RhoBAST, a RNA‐based PAINT probe, was developed from an RNA aptamer that binds with fast association and dissociation kinetics to a fluorogenic rhodamine (Figure 6C).^[^ ^139^ ^]^ A tetramethylrhodamine‐dinitroaniline (TMR‐DN) ligand remained bound to RhoBAST for 1.5 s, and subsequently dissociates and is replaced by a new ligand within ≈5 s. This process enables the molecule to display fast fluorescence blinking, which is a key requirement for SMLM. The RhoBAST:TMR‐DN systems support continuous and rapid fluorophore exchange, along with extremely high photostability and brightness. These systems were also the first fluorescent light‐up aptamers (FLAP) that were specifically designed for live‐cell SMLM. The RhoBAST:TMR‐DN systems permit subcellular and subnuclear RNA structures to be easily visualized in live or fixed specimens with excellent spatial precision (Figure 6C).^[^ ^139^ ^]^

### 4.4 Peptide PAINT

Peptide‐peptide interactions can be engineered for use in PAINT applications (Figure 6D). LifeAct, an actin‐binding peptide (17 amino acids in length), was coupled to Atto488 and used to reconstruct PAINT images of actin cytoskeleton with extremely high labeling density.^[^ ^140^, ^218^ ^]^ This approach was further applied to generate 18 peptide fragments of proteins that bind to cytoskeleton or focal adhesions and referred to as image reconstruction by integrating exchangeable single‐molecule localization (IRIS). The exchangeable IRIS probe allowed for sequential multitarget imaging of cytoskeleton and focal adhesions. Likewise, beginning with the 21 amino acid (aa) peptides of a E3/K3 coiled coil pair, adjustment of the peptide length tuned the affinity of the coiled coil to achieve even faster association kinetics (two‐fold) versus the classical DNA‐PAINT.^[^ ^141^ ^]^ The resultant peptide probe was successfully used in live cells to visualize microtubule and vimentin networks at super‐resolutions (Figure 6D).^[^ ^141^ ^]^

The use of peptide‐protein interactions for super‐resolution imaging inside of live yeast cells was recently introduced as LIVE‐PAINT.^[^ ^142^ ^]^ The key difference between LIVE‐PAINT and other PAINT methods based on dye, peptide and nucleic acid is that all of the components in LIVE‐PAINT are genetically encoded and expressed within the cell. LIVE‐PAINT used a short peptide sequence that was fused to the protein of interest (POI). Blinking was observed once the peptide reversibly bound to the FP‐fused protein binder. Peptide‐protein interactions are selected such that solution exchange occurs on a timescale that is shorter than or comparable to the bleaching lifetime, permitting the acquisition of several sequential images.^[^ ^142^ ^]^ LIVE‐PAINT also has a long data acquisition period that can extend over the duration which the target protein is expressed by using an inducible promoter, which allows LIVE‐PAINT to serve as a diagnostic of expression levels for expression optimization or regulation purposes. But limitations of this technique include the reliance on FPs with high spectral similarities, preventing simultaneous imaging of multiple target proteins. Moreover, the technique is constrained by the number of pairs of peptide‐protein interactions.^[^ ^142^ ^]^

### 4.5 Protein PAINT

The association and dissociation of fluorogen from fluorogen‐activating proteins (FAP) can support emitter blinking. FAPs often require the fluorogen to be supplied exogenously. Synthetic fluorogens enable rational generation of fluorescent molecules with desired characteristics. Also, in certain systems, the noncovalent nature of the interaction enables blinking of the fluorescent signal that is triggered by ligand binding and dissociation. For protein PAINT, mutants of bacterial lipocalin Blc, referred to as DiB (Dye in Blc), were engineered to diplay a substantial increase in fluorescence intensity upon binding of a cell‐permeable fluorogenic dye (M739).^[^ ^143^ ^]^ This strategy produced fluorescent signals with photostabilities that were an order of magnitude higher than FPs and enabled prolonged live‐cell super‐resolution microscopy based on PAINT and STED.^[^ ^143^ ^]^ Two spectrally distinct DiBs were engineered for two‐color PAINT imaging with a single fluorogen (M739).^[^ ^144^ ^]^ A split version of DiB was developed to address irreversible photobleaching by photoinduced decomposition of the M739 that result in the oxidation of amino acids side chains inside the protein‐ligand binding pocket. Split DiB exhibited improved single‐molecule brightness and applied to live‐cell protein‐PAINT imaging.^[^ ^145^ ^]^

UnaG is a natural fluorogen‐binding fluorescent protein that is derived from *Anguilla japonica*.^[^ ^219^ ^]^ UnaG produces oxygen‐independent green fluorescence that is selectively triggered by an endogenous ligand called bilirubin, which is a membrane‐permeable heme metabolite. This behavior enables facile control over kinetics, low background, and UV‐free reversible photoswitching (Figure 6E).^[^ ^146^ ^]^ On‐ and off‐switching rates are controlled by the concentration of bilirubin and the strength of the excitation light, while dissolved oxygen encourages off‐switching. The reaction mechanism for the photo‐oxidation of bilirubin in UnaG establishes that the absence of a ligand‐protein covalent bond permits the oxidized ligand to detach from the protein, which empties the binding cavity and allows a new ligand molecule to bind (i.e., blinking). SMLM of various subcellular compartments was performed from genetically encoded UnaG, further establishing this methodology as a facile approach for long‐term live‐cell and multicolor SMLM (Figure 6E).^[^ ^146^ ^]^

### 4.6 Exchangeable Probes for Coordinate‐Targeted Microscopy

The concept of PAINT can be repurposed for STED by using fluorophores that reversibly bind to their target and dynamically exchange with free fluorophores, which bypasses photobleaching. In STED, photobleaching limits the depletion intensity, thereby limiting the resultant resolution. Fast exchange kinetics, large fluorophore reservoirs, and high labeling density of the target structure are keys to obtain high‐quality STED images and to ensure long imaging times. Fluorogenic labels (e.g., Nile red and JF646‐Hoechst) that reversibly bind to their target structure were used to demonstrate whole bacterial cell, 3D, multicolor, and live‐cell STED microscopy (Figure 6F).^[^ ^125^ ^]^ Both Nile red and JF646‐Hoechst reversibly bind on their targets and have strong fluorescence intensities over multiple STED frames versus stationary‐labeled fluorophores. This strategy was extended to DNA‐labelled antibodies as a target‐specific approach for exchange‐based STED imaging.^[^ ^124^ ^]^ Cellular structures were labeled with target‐specific antibodies that include short DNA sequences used in DNA‐PAINT for transient binding to fluorophore‐labeled complementary oligonucleotides. Exchange‐based STED imaging benefits greatly from the ability to capture a large number of images at various axial locations, without the excitation or depletion laser compromising the fluorescence signal in the out‐of‐focus imaging planes.

Thioflavin T (ThT) is an exchangeable fluorophore for amyloid fibers and was used for STED imaging of amyloid fibers of mutant *α*‐synuclein (Figure 6G). This technique achieved a spatial resolution of 60–70 nm and improved photostability that supported long‐term STED imaging.^[^ ^126^ ^]^

### 4.7 Exchangeable Probes for DNA Imaging

The fluorescence emission strength of minor groove binders or intercalating DNA strains typically increases 100 to 1000 times following binding to the DNA.^[^ ^122^ ^]^ Some cyanines, including TOTO or YOYO, bind to DNA in a “bis‐intercalating mode,‘ which means that the aromatic portion of the cyanine intercalates while the amine ‘arms” lie in the minor groove. YOYO‐15 is a dimeric dye that intercalates between the base pairs along the DNA double helix. Internal rotational motion of the chromophores quenches fluorescence in solution. The chromophores become immobilized as a result of binding, and the quantum yield increases by ≈800 times. YOYO‐1 was exploited to image DNA molecules by blinking microscopy with MEA in the imaging buffer and by STED microscopy (Figure 6H).^[^ ^123^, ^220^ ^]^

## 5 Outlook

Fluorophores played pivotal roles ever since the advent of SRM. STED and SMLM concepts were introduced in the 1990s,^[^ ^14^, ^221^ ^]^ but these methods remained unrealizable until suitable fluorophores became available. Specifically, STED became practical after development in photobleaching‐resistant fluorophores with high efficiencies for stimulated emission.^[^ ^17^ ^]^ Likewise, SMLM first became possible^[^ ^23^, ^24^, ^25^ ^]^ after the discovery of photoswitching behaviors of GFP and Cy5.^[^ ^18^, ^21^ ^]^ RESOLFT and nonlinear SIM became usable developing FPs that exhibited reliable photoswitching behaviors that were reproducible over many photoswitching cycles.^[^ ^222^, ^223^ ^]^ New and improved probes continue to support breakthroughs in SRM and facilitate routes towards the ultimate goal of in vivo structural biology.

The spatial resolution of many SRM methods is theoretically unlimited.^[^ ^19^, ^31^, ^32^ ^]^ Yet it remains challenging to practically achieve molecular‐scale resolution at the sub‐10‐nm level. The resolution of SRM techniques approaches a few nanometers by the use of special localization schemes,^[^ ^216^, ^224^, ^225^ ^]^ fluorophores with unusual photostabilities,^[^ ^97^ ^]^ or precise correction of sample drift.^[^ ^216^, ^226^ ^]^ When the accuracy at which fluorophores can be localized to nanometer scales, the linkage error that characterizes the distance between the fluorophore and the molecule of interest will become a new limiting factor for resolution.^[^ ^227^ ^]^ Thus, the realization of SRM‐based structural biology will benefit from minimizing the probe size and the linkage between the fluorophore and the target site. As such, it would be best to improve the photostability of organic dyes without increasing probe size. Small binders for proteins such as nanobodies and SOMAmers have been applied to reduce the dye‐to‐target linkage.^[^ ^133^ ^]^ Ultimately, the direct attachment of dyes to the protein of interest can be realized by using genetic code expansion technology to incorporate a noncanonical amino acid with a functional group to the sequence of a protein of interest.^[^ ^228^ ^]^ Alternatively, the effective probe size can be reduced by physically swelling the sample volume.^[^ ^229^, ^230^, ^231^ ^]^ By this method, the linkage error of a pair of primary and secondary antibodies can be reduced from 15 to 7 nm after expansion.^[^ ^229^ ^]^ Likewise, large photostable probes such as nanoparticles and encapsulated dyes can have sub‐10‐nm linkage error after expansion.

The ultimate aim for SRM is to realize structural biology of live cells by resolving molecular structures in living cells. Revolutionary advances in cryo‐electron microscopy in recent years may soon enable in situ structural biology at atomic resolution.^[^ ^232^ ^]^ Yet, electron microscopy is incompatible with live cells due to the vacuum conditions and high energy of electron beams. Thus, SRM appears to be the only suitable technique to resolve ultrastructural dynamics in live cells. Yet, improving fluorophores for live‐cell SRM faces challenges with respect to labeling intracellular molecules in live cells, controlling on/off‐transitions in various intracellular environments, and minimizing damages to cell health. Currently, organic dyes require more facile, efficient labeling and reliable switching chemistries inside cells.^[^ ^233^, ^234^ ^]^ By comparison, FPs require better photon outputs and photostabilities.^[^ ^39^ ^]^ Alternatively, exchangeable probes permit fluorescence recovery after photobleaching, which extends observation time. Fluorogenic exchangeable probes indefinitely extend the observation window by exploiting a large reservoir of fluorophores without increasing the fluorescent background.^[^ ^139^, ^146^ ^]^

Advances in fluorophore photochemistry, instrumentation, and analysis, have enabled SRM techniques to support numerous discoveries of novel biological structures over the past decade.^[^ ^3^ ^]^ Further improvements in the photostabilities and other photophysical characteristics, such as photon budget and robust switching chemistries, of fluorophores may lead to new breakthroughs in realizing imaging techniques capable of molecular‐scale resolution in live cells.

## Conflict of Interest

The authors declare no conflict of interest.
